# Thrombotic Thrombocytopenic Purpura in Pediatric Patients

**DOI:** 10.3390/biomedicines13051038

**Published:** 2025-04-25

**Authors:** Niki Shrestha, Ebruphiyo Okpako, Robert W. Maitta

**Affiliations:** Department of Pathology, University Hospitals Cleveland Medical Center, Case Western Reserve University, Cleveland, OH 44106, USA; niki.shrestha@uhhospitals.org (N.S.); ebruphiyo.okpako@uhhospitals.org (E.O.)

**Keywords:** thrombotic thrombocytopenic purpura, congenital, immune-mediated, TTP, steroids, therapeutic plasma exchange, mutation, thrombocytopenia

## Abstract

Thrombotic thrombocytopenia purpura is a serious disease that can involve complex symptomatology, prolonged hospitalization, and a high risk of mortality if treatment is delayed. This disease is rare, but it is even rarer among pediatric patients. Even though it was first described 100 years ago, the earliest documented case was a pediatric patient. The last three decades have seen the discovery of the pathological mechanisms responsible for its clinical presentation. Symptoms/signs characteristic of microangiopathic hemolytic anemia with significant thrombocytopenia characterize the vast majority of patients. Its pathology centers on the accumulation of ultra-large von Willebrand factor multimers due to an enzyme deficiency that prevents their breakdown. Currently, in pediatric patients, two forms of the disease are known: congenital due to a mutation in the enzyme’s gene and immune-mediated due to enzyme depletion or neutralization secondary to autoantibody formation. With the advent of therapeutic plasma exchanges, immunosuppression, and, more recently, a TTP-specific nanobody, there is reason for optimism that the disease does not necessarily equate to a bad outcome. Thus, the aim of this review is to contrast the congenital and immune-mediated forms of the disease in pediatric patients while presenting them in the context of their pathologic mechanisms, diagnosis, and treatment.

## 1. Introduction

Thrombotic thrombocytopenic purpura (TTP) is a type of thrombotic microangiopathy (TMA) first described in 1924 by Dr. Eli Moschcowitz when he reported the case of a teenage girl who developed fever, pain, weakness, anemia, and petechial rash at the time of presentation [1]. However, no platelet count was available. A handful of schistocytes were observed in her peripheral smear. Two days after the initial symptoms, she developed neurological signs, and she passed away two weeks later. Autopsy results determined the presence of disseminated hyaline thrombi in the small vessels of the kidney, spleen, liver, and heart; however, medium-sized vessels were spared [1]. Although the first diagnosis of TTP was made, it remained uncertain whether this case was congenital (cTTP) or immune-mediated (iTTP) [2,3,4]. In the subsequent years the disease would be found to be very rare among pediatric patients, something that has made studies in this population particularly challenging.

TTP primarily involves the removal of platelets from circulation and secondary damage to erythrocytes, leading to thrombocytopenia and anemia. The disease is characterized by the formation of microthrombi in small blood vessels across all organ systems, resulting in severe complications, including renal failure and neurological deficits. Due to its rarity, cTTP is often misdiagnosed as iTTP, which can lead to significant lifelong morbidity and mortality. Therefore, its early identification and the prompt initiation of appropriate treatment minimize long-term complications [5]. Both cTTP and iTTP present as a Coombs test-negative TMA result with consumptive thrombocytopenia in the setting of various acutely affected organ systems with different degrees of severity [6,7]. It was in the latter part of the twentieth century that the pathologic mediators of the disease would be described. First the accumulation of von Willebrand factor (vWF) multimers and a later deficiency of the enzyme needed to cleave these multimers to prevent the seeding of platelets and the formation of microthrombi would be reported [8]. Similarly, mutations in the gene encoding the cleavage enzyme would be described soon after, and the subdivision between the congenital form and that which is immune in nature was cemented. Literature reviews have not fully contrasted these two forms of the disease in children and are limited because they were published prior to the approval of caplacizumab [2,9]. Therefore, in this article, we aim to outline the pathophysiology, symptoms across pediatric groups, and management options of cTTP while comparing and contrasting it to iTTP presentations in pediatric patients. The contrast of both forms of the disease will be emphasized so that greater awareness of up-to-date therapy options is presented in the context of each, especially since therapy may not be uniform across the two forms of the disease.

## 2. Congenital Thrombotic Thrombocytopenic Purpura

**Figure 1:**
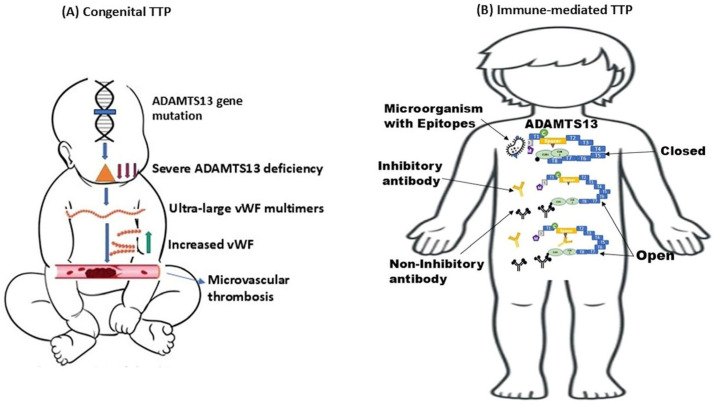
Pathophysiology of congenital and immune-mediated TTP. (**A**) Pathophysiology of cTTP. A mutation in the ADAMTS13 gene leads to absent or severely reduced enzyme activity, causing an accumulation of vWF multimers. This accumulation promotes excessive platelet adhesion and aggregation with subsequent microthrombi formation (adapted from [13]). (**B**) A model of an anti-ADAMTS13 autoantibody in iTTP. Under normal conditions, ADAMTS13 exists in a “closed” conformation, but autoantibodies, triggered by possible molecular mimicry with epitopes from pathogens, elicit cross-reactive antibodies that can either inhibit by binding to a private antigen in ADAMTS13 once it is in its open conformation or reduce the total concentration of the enzyme in circulation by binding to the CUB domain, thus leading to enzyme deficiency (adapted from [14]).

Upshaw–Schulman syndrome also known as cTTP is an extremely rare autosomal recessive disorder first described by Schulman in 1960 and later by Upshaw in 1978 that occurs with an annual incidence of fewer than one in a million individuals [2,5]. This condition arises from mutations in the *ADAMTS13* (a disintegrin-like and metalloprotease with thrombospondin type 1 motifs 13) gene, which encodes an essential protease responsible for cleaving vWF multimers [7,10], and specific mutations of this gene such as *D173G* result in severe cases of the disease [11,12]. The ensuing enzyme deficiency leads to the accumulation of ultra-large (UL) vWF multimers, which cause platelet aggregation and diffuse microvascular thrombosis, resulting in hemolytic anemia, thrombocytopenia, and organ ischemia (Figure 1A) [5,7]. Quantitatively, patients with low ADAMTS13 activity (<70% of normal) have a significantly higher incidence of stroke compared to those with higher activity levels, implying that the risk of thrombosis could also be elevated in heterozygous parents and siblings of individuals with cTTP [11]. As a result, it is important to examine for family consanguinity in patients with cTTP.

This diagnosis requires a high degree of suspicion and may present with numerous overt or clinically silent symptoms that involve almost any organ system with a variable frequency of episodes. The first episode typically occurs before the age of five, with an equal incidence in males and females [2]. The typical signs of cTTP include severe neonatal jaundice, often necessitating an exchange blood transfusion, along with recurrent childhood episodes of thrombocytopenia and microangiopathic hemolytic anemia (MAHA) that improve after fresh frozen plasma (FFP) infusions. Patients may also have milder symptoms during childhood, frequently presenting only as isolated thrombocytopenia, which can lead to the disease being overlooked by clinicians [5,15]. Disease episodes can be triggered by vaccinations, infections, and surgical procedures [2]. Early reports pointing to the effectiveness of plasma infusions in the treatment of cTTP also established that a subgroup of patients experienced frequent recurrences that were initially referred to as “chronic relapsing TTP.” This term was used to describe cTTP prior to obtaining a clear understanding of its underlying pathologic genetic mechanisms [11].

Disease presentation is characterized by clinical heterogeneity with progressive effects on the kidneys and brain [16]. The brain symptoms experienced by patients are neurological in nature such as headache, confusion, seizures, and other transient focal neurological deficits; renal dysfunction manifests by hematuria, proteinuria, and, in severe cases, renal failure; and the involvement of other organs, including cardiac, gastrointestinal, and pulmonary complications has also been reported [16]. About 50% of cases are so severe and chronic that they require monthly prophylactic plasma therapy (infusion), while the other 50% are less severe and experience prolonged remissions, thus not needing regular plasma infusions.

### 2.1. Pathophysiology and Genetic Alterations

Clinicians need to be aware that other etiologies can mimic TTP, but these mostly respond to distinct therapeutic approaches. For example, hemolytic uremic syndrome (HUS), a related condition, typically showing with severe renal failure and fewer neurological symptoms and often following an infection with a shiga toxin-producing strain of *Escherichia coli*, is frequently seen in young children. Likewise, TTP-like disorders arising from medications, bone marrow transplantation, pregnancy, HIV, and autoimmune diseases can represent a diagnostic challenge, but thankfully, these cases tend to be sporadic. Of note, when TTP presents, some patients may experience chronic relapsing symptoms, and rare familial or congenital cases present in neonates with frequent relapses [17,18,19,20]. In addition, recent data from mouse models have demonstrated that both genetic and environmental factors, alongside the lack of ADAMTS13, are essential for the complete manifestation of cTTP, while other unknown modulatory genes might still affect ADAMTS13 activity levels in patients with mutations [18].

ADAMTS13 is encoded by a gene located on chromosome 9q34 and is primarily synthesized in the liver by stellate cells and by vascular endothelial cells. The ADAMTS13 precursor consists of 1427 amino acids and features several structural components, including a metalloprotease domain and multiple thrombospondin type 1 (TSP1) repeats [11,18]. Its enzymatic target, vWF, is a multimeric protein produced by endothelial cells and megakaryocytes that facilitate platelet adhesion at sites of vascular injury by binding to platelet and connective tissue receptors. Under normal physiologic conditions, ADAMTS13 is the only known enzyme that cleaves vWF. Upon stimulation via shear stress, catecholamines, cytokines, or histamine, endothelial cells release UL vWF multimers that either stay attached to the cell surface or enter circulation [21]. A linkage analysis performed on four families with cTTP indicated that mutations in *ADAMTS13* caused a significant decrease in vWF cleaving activity in plasma, which is responsible for the development of TTP (Figure 1A) [20]. Without ADAMTS13, these UL multimers remain intact, resulting in excessive platelet adhesion and aggregation and diffuse thrombi formation [21,22]. In regard to the region of ADAMTS13 that mediates its enzymatic function, a study showed that select deletions of its catalytic domain significantly reduced its proteolytic activity over vWF, and the reintroduction of the spacer domain partially restored it, while adding more TSP1 motifs nearly returned its activity to that of the native full-length protein [19].

Mutations in *ADAMTS13* can be homozygous or compound heterozygous, which can result in either reduced production or increased clearance of the enzyme, leading to severe deficiency, an inability to cleave UL vWF multimers, platelet hyperaggregation, and consumptive thrombocytopenia [16]. To date, over 200 mutations, including missense, nonsense, frameshift, and splice site mutations, linked to cTTP have been identified and are linked to single families, but some of these mutations vary across different geographical regions and ethnic groups [5,7,16]. Most mutations have not been studied through in vitro mutagenesis and expression analysis; however, those that have been are characterized by defects in enzyme secretion. For example, some *ADAMTS13* mutants may be secreted but exhibit reduced or no enzymatic activity, resulting in a significant enzyme decrease in circulation, indicating that mutations affect not only biosynthesis but also proteolytic function [23]. Additionally, both in vitro and in silico studies have shown that select mutations negatively affect the function of ADAMTS13 or hinder its secretion [2]. As a result, cTTP should be considered in patients with severe ADAMTS13 deficiency, particularly in the absence of anti-ADAMTS13 autoantibodies [23].

*ADAMTS13* mutations have been shown to be specific to individual families, but some mutations occur with greater frequency in certain geographic regions [24]. Interestingly, it has been reported that there is minimal similarity in *ADAMTS13* sequence variations between Asian and European patients [25,26,27]. In European populations, two mutations, *p.R1060W* and the insertion *c.4143_4144dupA*, are frequently observed in cTTP patients and are commonly identified in large population studies. Research in Norway has indicated that *p.R1060W* appears in 0.3 to 1.0% of the population, while *c.4143_4144dupA* occurs in 0.04 to 0.3% [28]. Specifically, the *c.4143_4144dupA* mutation is primarily found around the Baltic Sea, central Europe, and Scandinavia, while *p.R1060W* has a broader geographic distribution [11]. This mutation is linked to residual ADAMTS13 activity, typically at 5 to 10% of normal levels in homozygotes, and is especially found in women diagnosed during their first pregnancy, frequently with severe preeclampsia in the second trimester [29]. Nonetheless, in the largest cohort studied, there was only a weak correlation between residual ADAMTS13 activity and age at diagnosis or cTTP severity [11]. Notably, in the International Hereditary Thrombotic Thrombocytopenic Purpura Registry, the *c.4143_4144dupA* mutation (exon 29; p.Glu1382Argfs * 6) was the most common and was found in 60 out of 246 alleles [27]. Likewise, an examination of residual ADAMTS13 activity and its association with disease onset while focusing on the carriers of the *c.4143_4144dupA* mutation found that homozygous status or residual ADAMTS13 activity did not significantly influence the disease phenotype [27]. Interestingly, compound heterozygotes with higher baseline ADAMTS13 activity tended to develop symptoms earlier.

It has been reported in one of the largest cTTP cohorts of pregnant women consisting of 16 women from two Bedouin families in southern Israel that all were homozygous for the same novel *ADAMTS13* variant linked to pregnancy complications [17]. Most reported cTTP cases (64%) were compound heterozygous, while 36% were homozygous. This study showed uniform homozygosity for the *c.3772delA ADAMTS13* variant, which allowed them to perform genotype–phenotype associations [17]. A large cohort of cTTP cases that consisted of 73 diagnosed cases in the UK, with 93% alive at the time of review, indicated that one-third of the patients had homozygous mutations, while two-third were compound heterozygous [30]. In this study, two peak times of presentation were noted: one being in childhood (median age: 3.5 years) and the other during adulthood (median age: 31 years). However, the presentations were often related to pregnancy. The study found that specific mutations, particularly pre-spacer ones, were more common in childhood onset cases. Furthermore, the most frequently observed mutation was the exon 24 *R1060W* missense mutation with a clear correlation between mutation location and age at presentation [30].

Studies have suggested an existing link between the location of *ADAMTS13* mutations and the associated ADAMTS13 activity levels. A study of 29 patients with cTTP found that those with residual ADAMTS13 activity < 3% experienced earlier disease onset [31]. This report noted that patients homozygous for the *R1060W* genotype, despite having higher residual ADAMTS13 activity, had their first TTP episode requiring treatment at a later age. These findings show the inconsistent genotype–phenotype correlations observed in cTTP. This is because alongside other intrinsic factors, disease severity can vary significantly among individuals, irrespective of the specific genetic mutations present [31]. Additional genetic or environmental factors are likely necessary for TTP development, with potential triggers including infections, pregnancy, surgery, heavy alcohol use, and certain medications. In addition, genetic factors related to the coagulation cascade, vWF, platelet function, and endothelial components may also play a role [18].

In some instances, deficiencies can be found in setting of multiple mutations. A young man was reported to have several episodes of TTP with undetectable ADAMTS13 activity caused by two novel mutations in the *ADAMTS13* gene: a single nucleotide deletion in exon 17 (*c.2042 delA*) causing a frameshift (K681C fs X16) and an additional missense mutation in exon 25 (*c.3368G > A*) resulting in p.R1123H [32]. Likewise, at least three distinct mutations have been identified in members of families with no relationship to one another. The *4143insA* mutation has been found in at least 15 patients who share a common haplotype in central-northern Europe, with over 25 polymorphisms identified in the coding sequence of *ADAMTS13*, and certain combinations of these polymorphisms or mixes of mutations and polymorphisms bring about a profound and significant deficiency in ADAMTS13 [33].

### 2.2. Clinical Presentations, Triggers, and Diagnosis

Diagnosing cTTP is challenging due to its rarity. Its clinical presentation varies considerably, with some individuals experiencing their first episode in infancy or childhood, while others may not manifest symptoms until adulthood, typically in their twenties or even thirties [34]. As mentioned earlier, children experiencing thrombocytopenia and jaundice may go unrecognized or may be misdiagnosed for years. Consequently, a high level of suspicion is essential for all patients with acute TMA, regardless of age [23]. This is because the disease could first manifest in the neonatal period as prolonged jaundice, with 40–45% of affected infants requiring exchange transfusions [35]. It is often misidentified as the more prevalent ABO incompatibility-associated hemolysis, which typically leads to severe hyperbilirubinemia. As a result, the initial hours of life are crucial for infants with cTTP, since turbulent blood flow in the patent ductus arteriosus could lead to the uncoiling of UL vWF multimers, facilitating platelet binding and aggregation and resulting in a systemic microvascular thrombosis [36]. Thankfully, before birth complications are exceedingly rare due to turbulent circulation not occurring in the fetus [15]. Along these lines, a study on 37 cTTP patients, focusing on nine women diagnosed during their first pregnancies, found that six had childhood thrombocytopenia misdiagnosed as idiopathic or immune thrombocytopenia (ITP). Their pregnancies were complicated by thrombocytopenia, with many resulting in stillbirth or premature birth, emphasizing the need for ADAMTS13 activity testing in childhood and during pregnancy-associated thrombocytopenia [37]. In older infants, symptoms often arise following infections or trauma that can be confused with conditions like HUS or Evans syndrome, which are similarly characterized by signs of autoimmune hemolytic anemia and thrombocytopenia [35].

Delay in cTTP diagnosis can result in severe and long-lasting thrombocytopenia and damage to vital organs, potentially leading to infant mortality shortly after birth [38]. A study from Japan revealed that 42% (18 out of 43) of cTTP patients received neonatal exchange transfusions for severe jaundice, yet only 4 (9.5%) of these patients were diagnosed with cTTP within the first 6 months of life [15]. Similarly, a study from Norway found that 45% (9 out of 20) of cTTP patients had undergone exchange transfusions for jaundice, with the diagnosis of cTTP being delayed until after their first year [28]. A larger analysis of 226 cTTP patients revealed that most were not timely diagnosed, particularly males and nulliparous females [39]. Severe jaundice at birth was noted in 83 of these patients, but only 3 (1.3%) received timely treatment. Among those who survived infancy, 34% experienced major morbidities, including strokes, often at a young age. Unsurprisingly, survival rates were lower than in the general population [39]. Therefore, the timely identification and treatment of cTTP in newborns are paramount since it can result in significant morbidity and early mortality [38].

In adults, heavy alcohol consumption may trigger acute TTP episodes, particularly in men. Additional potential triggers include inflammatory conditions, trauma, and certain medications. In this patient group, symptoms include signs of MAHA with thrombocytopenia, along with neuro-psychiatric symptoms such as headaches, difficulty concentrating, and depression, which can affect over 60% of patients and contribute significantly to the disease burden [27]. Myocardial infarction remains rare; however, renal failure can develop with age; according to the international registry, 10% of patients required renal replacement therapy, and in more severe cases, 2% needed renal transplantation [27,40]. In some rare instances, patients may present much later in adulthood with severe thrombocytopenia and MAHA signs, leading to a suspicion of acute iTTP rather than cTTP [41]. In this study, despite effective initial treatment resulting in platelet count normalization, patients experienced relapses, prompting further genetic evaluations. Importantly, patients had a history of multiple bleeding events, neurological issues during pregnancy, and complications from antiviral therapies, raising the suspicion for unrecognized cTTP [41]. Laboratory tests showed minimal ADAMTS13 activity and antigen levels, and cTTP diagnosis was confirmed with a homozygous missense mutation (*p.Ile143Phe*) in the *ADAMTS13* gene, illustrating that a thorough clinical evaluation is important to identify the correct diagnosis in such patients [41].

Thus, it should not be surprising that other diagnoses like primary ITP are entertained during childhood but were later found to have been cTTP based on additional symptoms/signs of dyspnea, fatigue, fever, and jaundice [42]. Laboratory test results consistent with MAHA in the setting of absent ADAMTS13 activity should be suggestive of a diagnosis of cTTP rather than one of secondary TTP, even in the presence of contemporary infections. The use of antibiotics and therapeutic plasma exchange (TPE) can lead to clinical improvements in such settings, thus allowing the symptoms to be managed with biweekly FFP infusions and prophylactic antibiotics, which demonstrates that addressing the underlying trigger reduces the need for greater plasma exposure while controlling MAHA signs [42]. Pregnant patients diagnosed with cTTP also face increased risks of fetal and maternal complications, including preterm birth, preeclampsia, stillbirth, and other severe pregnancy-specific health issues. As a result, patients with cTTP during pregnancy can experience fetal demise, highlighting the importance of the timely recognition of the condition and distinguishing it from other syndromes like preeclampsia and HELLP syndrome [43]. Interestingly, the use of platelet imaging and proteomics in such cases could point to molecular interactions that contribute to the complications associated with cTTP during pregnancy.

### 2.3. Laboratory Workup

Diagnosing cTTP requires a comprehensive approach that involves clinical, laboratory, and genetic assessments. Blood and morphologic tests can reveal signs of hemolytic anemia (low hemoglobin, elevated reticulocyte count, and the presence of schistocytes on a peripheral smear) and thrombocytopenia. The ADAMTS13 activity assay typically shows significantly reduced or undetectable enzyme activity. An ADAMTS13 inhibitor test helps distinguish between congenital and immune-mediated forms of TTP, with inhibitors usually absent in cTTP. Importantly, genetic testing is essential and is the standard for identifying mutations in the *ADAMTS13* gene, thereby definitively confirming the diagnosis (Table 1) [5].

#### 2.3.1. ADAMTS13 Activity Assay/Anti-ADAMTS13 Autoantibody

There are two key steps involved in ADAMTS13 measurements, which include first incubating test plasma with a vWF substrate and allowing the enzyme to cleave it, followed by the detection and quantification of the cleavage products to determine activity levels. This is known as the fluorescence resonance energy transfer test (FRET), which detects and measures the cleavage products of a synthetic vWF peptide [44]. Moreover, detection methods can be direct (measuring cleavage products) or indirect (measuring residual vWF). In addition to FRET, chromogenic assays and collagen-binding assays can also be utilized. Results are reported as a percentage of normal pooled plasma with detection limits below 1% to 5%, and ADAMTS13 activity assay scores that are <10% of the normal score are considered diagnostic of TTP [45].

#### 2.3.2. ADAMTS13 Functional Inhibitor Assay

Once severely deficient ADAMTS13 activity is confirmed, testing for the presence of antibody inhibitors against ADAMTS13 is undertaken. If positive results are obtained, an inhibitory (also known as Bethesda) assay is performed to establish the inhibitory nature of the antibodies detected [46]. These tests will establish the presence of neutralizing autoantibodies through a 1:1 mixing of patient’s and normal plasma or through pre-determined serial dilutions [35]. If an inhibitor is found, a diagnosis of iTTP is likely, and this represents the most direct approach to differentiate it from cTTP. ADAMTS13 autoantibodies, primarily anti-ADAMTS13 IgG, can also be detected using commercially available ELISA kits [46]. Nevertheless, available assays only detect free autoantibodies, not those bound to ADAMTS13 in immune complexes [47].

#### 2.3.3. Extended Platelet Profiles

ADAMTS13 testing is often sent to reference laboratories, which can delay results and often require TPE initiation before results are available. However, the measurement of immature/reticulated platelets using fluorescent-capable hematology analyzers can help increase the suspicion of the disease while waiting for ADAMTS13 results. Our group found that this young platelet population is uniformly below the reference range in patients with new-onset iTTP. High absolute immature platelet counts (A-IPCs) characterize compensated consumptive processes, while depressed production could be indicative of a lack of compensation or bone marrow unresponsiveness [48]. Notably, A-IPC hypoproduction predicts ADAMTS13 deficiency with great specificity, and fold increases in these immature counts correlate with therapy responses [49,50]. In pediatric iTTP patients, A-IPC levels at admission have also been predictive of ADAMTS13 deficiency, but the subsequent instability in A-IPC increments characterized therapy responses which required prolonged therapy, and was uniformly observed in this patient group prior to the stabilization of mature platelet counts [51]. However, it remains to be determined if these counts will be distinct in the setting of cTTP.

#### 2.3.4. Disease Scoring

To quickly assess the likelihood of ADAMTS13 deficiency, scoring systems such as the French and PLASMIC scores have been derived to assist in clinical decision making [52,53]. In high-risk groups, the likelihood of severe ADAMTS13 deficiency (activity < 10%) is predicted to be 94%, with 2 points on the French scale and 62–82% with 6–7 points on the PLASMIC one. Both models are intended to predict ADAMTS13 deficiency in patients with suspected TTP and are not to be used in asymptomatic individuals [54]. However, there are limitations in the utility of the PLASMIC score, as it can overstate or underestimate the risks of iTTP in some patients [49,51], and it is of limited utility in pediatric populations [55].

### 2.4. Treatment

The primary treatment for cTTP involves plasma replacement therapy to provide functional ADAMTS13 enzymes. With appropriate treatment, most cTTP patients can lead relatively normal lives. The regular monitoring of ADAMTS13 activity and clinical status is essential to prevent acute episodes and manage complications. Lifelong therapy may be necessary, and genetic counseling is recommended for affected families.

#### 2.4.1. Therapeutic Plasma Exchange (TPE)

The first-line treatment for TTP for the last two decades has been TPE performed daily using ABO type-specific FFP as a replacement fluid. However, this therapy is favored for patients with iTTP and not cTTP. This therapy supplements depleted ADAMTS13 and removes circulating anti-ADAMTS13 autoantibodies, as well as other soluble mediators that are indicative of the disease. The timely initiation of TPE is critical to prevent early mortality, and typically, 1–1.5 times the plasma volume is exchanged per procedure [50]. Importantly, there is no set duration or number of procedures, and treatment continues until a sustained clinical response (defined as a normal platelet count) is achieved for two consecutive days; however, in the setting of cTTP, if TPE is performed, few procedures are required, since the goal is to restore the missing enzyme to physiologic levels.

Thus, TPE is not recommended for all patients. A study of 60 patients with TMA, categorized based on ADAMTS13 activity levels, indicated that patients with mild enzyme deficiency could be managed without TPE, supporting the idea that ADAMTS13 activity is not only a useful diagnostic test but also, in this setting, a guide to therapy [56]. FFP given to cTTP patients should be collected from the fewest number of donors possible to reduce the risks of allergies, anaphylaxis, disease transmission, and other adverse reactions associated with infusion. Antihistamines and corticosteroids may occasionally be used to prevent allergic reactions to FFP, although there is limited scientific evidence to back these practices. The recommended FFP volume administered to patients is typically 5–10 mL/kg every 2–3 weeks, with an initial volume of 10 mL/kg during onset, and the transfusion duration should be adjusted based on platelet counts and patients’ clinical responses [47,54]. In 2018, the International Society on Thrombosis and Hemostasis (ISTH) established a multidisciplinary panel to develop treatment recommendations for iTTP and cTTP using the Grading of Recommendations Assessment, Development, and Evaluation approach [41]. The expert panel recommended administering 10–15 mL/kg of FFP every 1–3 weeks [54,57]. For patients with cTTP in remission, the ISTH panel suggested either plasma infusion or a watch-and-wait approach, based on very low certainty evidence. The panel emphasized that no clear recommendation could be made, and decisions should be guided by individual patient preferences and clinical circumstances [57].

In cases of refractory TTP or worsening organ damage, more frequent TPE may be considered. While determining the efficacy of TPE alone can be challenging due to concurrent therapies, there has been no significant efficacy differences found between available plasma replacement products, i.e., FFP and cryopoor plasma [58,59]. The latter was, at one point, thought as being more therapeutically effective; however, a randomized controlled trial indicated that its efficacy was similar to FFP [60].

#### 2.4.2. Immunosuppression and Emerging Therapies

Traditionally, corticosteroids have been used for immunosuppressive therapy. Current treatments also include rituximab, a humanized monoclonal anti-CD20 antibody shown to be effective in newly diagnosed, refractory, and relapse-prone cases [61]. However, these agents are used and shown to be effective in iTTP for obvious reasons. In cases that are refractory/unresponsive to rituximab, other anti-CD20 therapies such as obinutuzumab and ofatumumab can be used, which, by targeting different epitopes of CD20, may prove therapeutically useful and still achieve B-cell suppression [62,63].

A new drug, caplacizumab, an anti-vWF nanobody that blocks platelet binding to vWF and prevents microthrombi formation, is the first approved drug that is specific for TTP [47,61]. The TITAN (phase II) and HERCULES (phase III) trials revealed that caplacizumab, when used alongside TPE, reduced the length of hospitalizations, the number of TPE sessions, and the time to a treatment response, improving organ function and survival in iTTP [64,65]. It has a favorable safety profile, with manageable bleeding events, and has been approved by government regulatory agencies in Europe and the United States [66]. Furthermore, it has been shown to be safe even in very young iTTP patients without adverse events [67,68]. It will be of interest if this therapy will also be approved for routine use in cTTP patients.

#### 2.4.3. Recombinant Human ADAMTS13 Therapy

The development of recombinant ADAMTS13 (rADAMTS13) offers a potential new treatment to expand existing therapies for ADAMTS13 deficiency. It has shown promise in vitro as a therapeutic agent for congenital ADAMTS13 deficiency [61]. However, in iTTP, circulating anti-ADAMTS13 autoantibodies may hinder rADAMTS13, limiting its effectiveness. Nevertheless, it is encouraging that a study looking at iTTP patients found that high concentrations of rADAMTS13 overcame the presence of these autoantibodies and restored enzyme activity [69]. In 2023, the United States’ Food and Drug Administration approved a purified rADAMTS13 agent, named Adzynma, to supplement the deficient enzyme in patients with cTTP. It was shown to be safe and effective and was approved for prophylactic use and on-demand enzyme replacement therapy for acute disease episodes [70]. It can be administered intravenously every two weeks, while for on-demand treatment, it is given once daily [70].

A phase I study that evaluated the safety, tolerability, and pharmacokinetics of rADAMTS13 (BAX 930) in patients with severe congenital ADAMTS13 deficiency found that it was well tolerated, with no serious adverse events or formation of anti-ADAMTS13 antibodies [71]. Dose-dependent increases in ADAMTS-13 antigens and activity were observed, peaking within 1 h; and higher doses led to persistent ADAMTS13-mediated vWF cleavage and reduced vWF multimeric size, with a pharmacokinetic profile comparable to plasma infusion [71]. Subsequently, a phase III, randomized, controlled trial assessing the efficacy and safety of rADAMTS13 in comparison to standard treatment found that patients receiving rADAMTS13 for prophylaxis experienced no acute TTP events, had a minimal number of TTP symptoms, and achieved nearly 100% of normal ADAMTS13 activity levels [72]. This agent is anticipated to offer significant benefits for cTTP patients, as it may be safer than plasma infusion and TPE, and may potentially lead to better efficacy and sustained responses.

#### 2.4.4. Gene Therapy

Gene therapy for TTP is still an emerging area of research, but it holds potential in patients with cTTP, where a functional copy of the ADAMTS13 gene would likely restore enzyme function. Research in both preclinical and clinical settings has shown that human rADAMTS13 enzyme replacement therapy is a safe and effective treatment for TTP [73]. Encouraging results from a mouse model of shiga toxin-induced TTP have indicated that *ADAMTS13* gene transfer has the potential to treat cTTP (Table 1) [74]. While gene therapy for cTTP remains experimental, it holds promise for long-term disease management, especially for patients who have severe, recurrent, or treatment-resistant forms of the disease. Nevertheless, challenges like ensuring the safe, specific, and efficient delivery of the gene while avoiding immune reactions due to protein expression still need to be addressed.

## 3. Immune-Mediated Thrombotic Thrombocytopenic Purpura

Pediatric onset TTP represents approximately 10% of all TTP cases. It is considered pediatric TTP when the initial episode occurs before the age of 18 [2]. iTTP occurs in approximately 1 case per 1 million children [2]. The incidence of the first episode of iTTP is seen more frequently in girls compared to boys, and the ratio of occurrence is 2.5F:1M. The age distribution at presentation can range from 4 months to 17 years, with a median age of 13 years [2,3,4]. Notably, others have reported that the incidence in females vs. males may be much greater and, in some cases, be as high as 4:1 [75]. For example, an autopsy analysis of 25 patients with TTP lends further support that the disease occurs more frequently in females [76].

The Oklahoma TTP-HUS registry, which has recorded over two decades worth of data of iTTP patients, including adults and children, has reported an annual incidence of TTP in children of 0.09 cases per 1 million, which is 3% of the incidence rate in adults of 2.88 cases per 1 million [77]. It also reports the disease being more common among girls in a similar proportion seen among adult females. However, according to this registry, disease presentation is more common among older children [77]. A study of 45 children with iTTP showed that the median age at presentation was 13 years, ranging from 7 to 16 years and with an F:M ratio of 2.5:1 [78], further confirming the higher prevalence of TTP in females.

### 3.1. Pathophysiology of iTTP

Its pathophysiology was first described in the early 1980s when the presence of UL vWF multimers in the plasma of TTP patients was detected, which later became undetectable when remission was achieved [79]. Over a decade later, it was reported that disease presentation was due to the deficiency of a UL vWF-cleaving protease, which was absent in patients with cTTP and deficient in patients with iTTP [80]. Soon after, the presence of autoantibodies as the causative trigger for the absence of protease activity was confirmed [81]. At one point, it was proposed that a symptomatic “pentad” characterized disease presentation, which included fever, hemolytic anemia, thrombocytopenia, acute renal failure, and neurologic signs. Nowadays, it has become quite evident that this is not the case since the absolute majority of cases do not present with symptoms that fit all of these categories; instead, signs of MAHA with thrombocytopenia are considered characteristic of the disease [57]. Finally, some have favored subdividing iTTP into primary, in which there may not be precipitating causes, and secondary, in which a precipitating insult leads to the disease [75,82]. However, this may not be fully supported by the current data. Both T and B cells are involved in the formation of antibodies in patients with iTTP through the recognition of linear peptides and conformational epitopes on the exposed surfaces of the protein. Subsequently, the activation of T cells through binding to T-cell receptors and a number of co-stimulatory signals initiates a sequence of events that lead to cytokine release and B-cell activation [8].

#### 3.1.1. ADAMTS13 Activity

UL vWF is stored mainly in the Weibel Palade bodies of endothelial cells and secreted into circulation, as large multimers have several functions, including maintaining primary hemostasis, promoting platelet adhesion, and mediating aggregation by binding to platelet glycoproteins (GP) 1b-IX and GPIIbIIIa [83]. As mentioned earlier, ADAMTS13 is required for vWF cleavage, and autoantibodies against the enzyme lead to its inhibition and clearance from circulation, thus triggering the accumulation of vWF multimers [84]. ADAMTS13 is made up of multiple domains, which include the metalloprotease (M) domain, the disintegrin (A) domain, the first thrombospondin type 1 (TSP1-1) repeat, a cysteine-rich (C) domain, a spacer (S) domain, seven more thrombospondin type 1 repeats, and two CUB domains (complement C1r/C1s, sea urchin epidermal growth factor, and bone morphogenetic protein) [85]. An enzyme activity of <10% is considered diagnostic; however, in those patients with borderline ADAMTS13 activity (10–20%), the diagnosis of iTTP can be challenging. Two ADAMTS13 conformations have been identified: one is open, and the other one is folded/closed [86]. The folded or closed conformation is the inactive enzymatic form and is normally characterized by the central S domain interacting with the C-terminal CUB domain [87]. The open conformation, on the other hand, which is observed in patients with iTTP, shows a disrupted S-CUB interaction with an exposed cryptic epitope in the S-domain that triggers antibody formation (Figure 1B) [87,88,89].

#### 3.1.2. Anti-ADAMTS13 Autoantibodies

Patients with iTTP have IgG antibodies that can bind to the spacer domain and mediate its inhibition, and these IgGs are readily detectable in relapsing patients. Of note, there is a 3.6 times higher risk of relapse in patients with both severe ADAMTS13 deficiency and anti-ADAMTS13 antibodies. On the other hand, a higher relapse risk is seen in younger patients with lower ADAMTS13 activity, whereas ADAMTS3 antibody IgG levels may not be predictive of relapse [2,90]. Anti-ADAMTS13 autoantibody epitope mapping in patients with iTTP has shown the presence of a polyclonal immune response, in which the majority of patients develop autoantibodies against the cysteine/spacer (CS) domain (Figure 1B) [88]. These anti-ADAMTS13 autoantibodies can be either neutralizing, which inhibits enzymatic function, or non-neutralizing, in which the enzyme concentration is markedly affected by their presence [91]. Lower ADAMTS13 antigen levels have been reported in patients with autoantibodies against both the N- and C-termini compared to patients who had only N-terminus antibodies [92]. It has also been reported that there is a strong association between ADAMTS13 antigenic depletion at presentation and disease severity [93]. Furthermore, a significantly low enzyme concentration at presentation has been associated with fatal disease outcomes, and a five-fold higher rate of mortality is observed in patients with ADAMTS 13 antigen levels in the lowest quartile [93]. Thus, an open ADAMTS13 conformation can be used as a biomarker for making the diagnosis [92]. The free anti-ADAMTS13 IgG subclasses observed during the acute phase of the disease and remission are mainly IgG1 and IgG4, without subclass switching being observed; however, IgG4 represents the predominant subclass detected during both the acute phase and during remission [94]. IgG4 is the least effective subclass of the IgGs since it is unable to activate the complement system via the classical pathway. IgG4 has high affinity for FcγRIA as a free monomer; however, in immune complex formation, it binds with low affinity to FcγRIIA, FcγRIIB, and FcγRIIIA [94].

#### 3.1.3. Immune Complexes

The formation of circulating immune complexes (CICs) has been implicated in the pathophysiological mechanism of iTTP. These complexes are also implicated in the pathophysiology of other autoimmune diseases. These CICs have been hypothesized to either directly affect the enzymatic activity of ADAMTS13 or cause severe deficiency through a mechanism still not defined [95]. Some of the properties of CICs include complement and leukocyte activation, which trigger cellular damage and inflammation typically seen in autoimmune diseases [95]. Along these lines, a study revealed that 39–93% of patients with iTTP presenting in the acute phase have immune complexes, with C3a, C5a, and factor Bb found to be elevated above baseline [47]. This elevation of C3a and C5a suggests that there is an activation of the complement system through the classical pathway [96], whilst the elevation of factor Bb indicates the activation of the alternative pathway. Nevertheless, the activation of the complement system by immune complexes typically occurs via the classical pathway, which leads to the activation of C3 and the subsequent formation of C3a [97]. The complement system is important to both the innate and adaptive immune responses, as well as angiogenesis, the breakdown and clearing of immune complexes, lipid metabolism, and pathogen recognition and elimination [98]. The detection of IgG–immune complexes post-remission may be indicative of steady low-level production of autoantibodies that, together with autoreactive follicular dendritic cells, maintain the suboptimal levels of ADAMTS13 [94]. Of note, the activation of the complement system in acute iTTP episodes can occur via immune complexes or infected/damaged cells. Moreover, patients with the highest C3a levels produced IgG subclasses that were associated with higher complement activation [96]. Lastly, the comparison of the relationship between UL vWF multimers and complement activation showed that an elevated platelet count, lactate dehydrogenase (LDH), and C4d were likely to decrease the concentration of multimers, while increased SC5b-9, C3a, and C5a had the opposite effect [99,100].

### 3.2. Human Leukocyte Antigens (HLAs)

T-cell recognition of ADAMTS13 epitopes requires its presentation within MHC class II of antigen-presenting cells [8,101]. HLA alleles are thought to be involved in the development of autoimmune diseases, likely due to the recognition of self-induced peptides by low-affinity CD4^+^ T cells that escaped negative selection in the thymus [102,103]. Among these, the intrinsic stability of HLA-DQ, which is poorly expressed on cell surfaces, has been linked to the onset of autoimmunity [104]. Studies have indicated that in Caucasian patients, the expression of HLA DQ-7, HLA DRB1 * 11, and HLA DRB3 * may increase the risk of developing iTTP [82]. Reports have also indicated that there is an increased frequency of HLA-DQB1 * 05:03 in patients with iTTP, and this could be the result of this HLA’s inability to present effectively immune-dominant peptides, leading to defective negative selection [105]. Furthermore, the *rs6903608* HLA gene variant has been reported to be a risk factor for both iTTP during acute presentation and relapse [104,106]. A study from the French registry of 191 patients with a confirmed iTTP diagnosis showed an increased disease risk among Caucasians with HLA-DRB1 * 11, while HLA DRB1 * 04/DR53 had a possible protective role [107]. On the other hand, when looking at the role of HLA among individuals of African descent, researchers found that despite some in the cohort having increased susceptibility for autoimmune diseases, when diagnosed with iTTP, they had approximately four times lower mortality compared to Caucasian patients, even in the setting of low expression of the protective allele HLA DRB1 * 04 [108,109]. This indicates that other factors, some of which could be related to other genes, play a role not only in disease occurrence but also potentially in iTTP severity.

### 3.3. Clinical Presentation and Triggers

**Figure 2:**
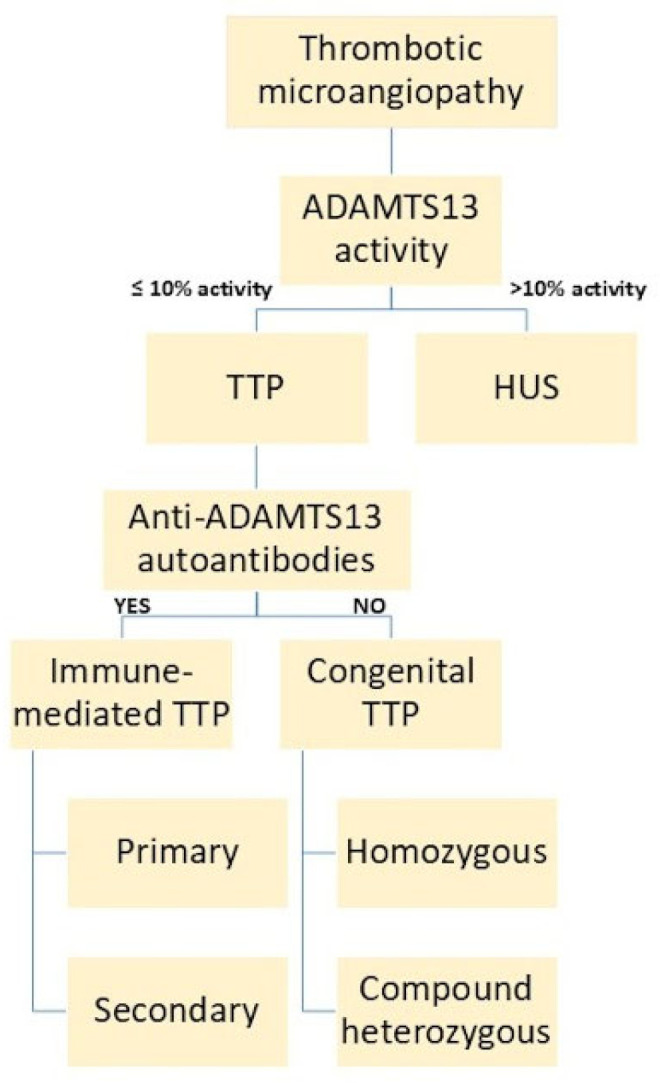
Diagnostic decision tree for patients with thrombotic microangiopathies. ADAMTS13 deficiency will characterize TTP diagnosis and the presence of autoantibodies to the enzyme, increasing the likelihood that the presentation is not cTTP but iTTP.

Primary iTTP is, by definition, an autoimmune disease since there are no underlying known triggers, and it is brought about by the formation of an autoantibody to a self-protein. As mentioned earlier, finding all elements of the pentad is unlikely since the significant majority of patients may have symptomatology and findings that are non-specific. However, MAHA signs and thrombocytopenia are seen in the majority of patients presenting with new-onset iTTP [3]. These patients have ADAMTS-13 activity of <10% in the presence of ADAMTS-13 autoantibodies (Figure 2). On the other hand, the hypothesized secondary iTTP autoantibodies against ADAMTS13 are related to the presence of underlying autoimmune conditions (e.g., systemic lupus erythematosus, Sjogren’s syndrome, and rheumatoid arthritis), infections (e.g., CMV and HIV), hormone fluctuations (e.g., pregnancy), and even medications (e.g., ticlopidine, quinine, simvastatin, trimethoprim, and pegylated interferon) [82]. A study of almost 100 pediatric patients with iTTP from over 20 children’s hospitals found that the majority of patients (59%) had no underlying comorbidities; however, the remainder had comorbidities such as rheumatoid arthritis, cancer, bone marrow, and solid organ transplant, as well as vitamin B12 deficiency, with some having more than one comorbidity at the same time [110]. Similar to infections, vaccines can result in undesired immune responses, leading to the development of autoimmune diseases. Possibly, vaccine antigens elicit an antibody response that cross-reacts with ADAMTS13, which is capable of neutralizing the enzyme [111]. For example, COVID-19 infection is suspected to induce antibody-mediated platelet apoptosis via an unknown mechanism, resulting in thromboembolic events [112]. Notably, reports have indicated that some patients who were vaccinated against COVID-19 developed iTTP as a result of it [113,114]. However, even though this may suggest that the vaccine is suboptimal, this is not unexpected, since any vaccine with a given antigenic specificity can elicit an undesired reaction in a recipient who has the gene predisposition or susceptibility to have such responses.

### 3.4. Laboratory Diagnosis

MAHA is the highlight of the clinical presentation of iTTP. This is evident by the presence of schistocytes on peripheral smears with markers of hemolysis such as elevated LDH secondary to platelet consumption and damage to erythrocytes, increased unconjugated bilirubin, and decreased haptoglobin [54,82]. Anemia in iTTP patients usually presents with hemoglobin concentrations of 8–10 g/dL [45,54]. Patients with iTTP can also have a higher percentage of schistocytes (4–8%), compared to patients with other TMAs (0.2–2%) [45]. More importantly, iTTP patients’ thrombocytopenia can be severe, with most having platelet counts as low as 10–17 × 10^9^/L [45,54,92]. ADAMTS13 activity testing is considered the gold standard for iTTP diagnosis. Thus, the evaluation of ADAMTS13 activity (<10%), the antigen concentration, possibly its conformation, and antibody presence (inhibitor) are important in making the diagnosis of iTTP [45,47]. As mentioned earlier, ADAMTS13 activity in the range of 10–20% requires additional findings such as testing for an open ADAMTS13 conformation to support an iTTP diagnosis [92,115]. An activity of >20% is unlikely to be iTTP [35]. Finally, the presence of anti-ADAMTS13 autoantibodies differentiates iTTP from cTTP (Table 1 and Figure 2) [45,92].

### 3.5. Treatment

#### 3.5.1. Therapeutic Plasma Exchange

TPE represents the most common therapy used to treat patients presenting with iTTP. According to the American Society for Apheresis (ASFA), TTP is a category I indication for apheresis [116]. What this translates into is that TPE is considered a first-line therapy approach for a patient with iTTP. These procedures are performed by exchanging 1–1.5 plasma volume daily using ABO-type-specific plasma until platelets achieve a count of 150 × 10^9^/L for two consecutive days [50]. More importantly, as per ASFA guidelines, other forms of TMA include lower indications due to the limited or lack of response to TPE.

#### 3.5.2. Immunosuppression

Immunosuppressive agents such as glucocorticoids are used in conjunction with TPE in the management of iTTP, with the goal to suppress anti-ADAMTS13 autoantibody formation [54]. Although there are no clinical trials to back up this combination vs. TPE as a standalone therapy, the autoimmune nature of iTTP supports its use [47]. Case in point, in a study comparing prednisone to cyclosporine as an adjunct therapy to TPE, it was shown that ADAMTS13 autoantibodies were significantly reduced in patients receiving steroids, who also experienced an earlier increase in ADAMTS13 activity compared to those receiving cyclosporine [117]. Regarding stronger immunosuppression, rituximab, a chimeric monoclonal antibody that targets the CD20 receptor and presents on the surface of B cells, has been shown to be highly effective in the setting of iTTP [118]. In those patients with the disease that has been shown to be difficult to treat and thus considered refractory, rituximab has resulted in improvements in platelet counts and longer relapse-free time periods [119]. Importantly, in those few patients shown not to respond to rituximab, other anti-CD20 antibodies such as ofatumumab and obinutuzumab with specificity for other epitopes in the CD20 molecule have shown marked inhibition in autoantibody production, sustained normalization of ADAMTS13, and recovery from the disease [62].

#### 3.5.3. Caplacizumab

Caplacizumab is a newly approved medication specific for iTTP treatment to be used in combination with TPE and is found to elicit platelet recovery with shorter hospital stays [64,65]. It is a humanized anti-vWF therapy with specificity for the A1 domain, through which it interferes with the interaction between the GPIb-IX-V receptor on platelets and vWF [120,121]. Caplacizumab has not been approved for use in pediatric patients; however, there have been cases of off-label use for the management of pediatric iTTP, which showed that it was safe and led to clinical improvements [67]. In this regard, model-based stimulation for pediatric dosing guidance for caplacizumab recommends doses of 5 mg if the patient’s body weight is <40 kg and 10 mg if the body weight is >40 kg [122]. A report of three young iTTP pediatric patients managed with immunosuppression who received daily TPE and caplacizumab showed that the platelet response occurred as early as after the loading dose or the first dose, reaching normal platelet counts by the fourth day of treatment [123]. Notably, the normalization of ADAMTS13 activity and the disappearance of its inhibitor occurred within several weeks [123]. A study of sixteen minors showed that patients treated with caplacizumab for a total of 31 days at doses of 10 mg daily for those weighing >40 kg and 5 mg for patients weighing < 40 kg in combination with steroids and TPE led to a full response and platelet count normalization by day 3, as well as the normalization of ADAMTS13 activity by day 35 [124]. Hence, despite clinical trials not being performed in pediatric iTTP patients, it is reassuring based on these data that the medication is safe and effective in pediatric patients.

## 4. Conclusions

TTP in children remains a rare presentation such that if suspected in a young patient, it remains not only a therapeutic challenge but one in which constant and obstinate vigilance is needed to not miss a disease which in either its congenital form or immune in nature can be associated with serious and possibly life-long complications. Therapy should not be delayed if this disease is part of the differential diagnosis and ought to be initiated empirically if necessary. ADAMTS13 deficiency/absence will confirm the diagnosis, and the presence of an inhibitor or the presence of an antibody with the concomitant depletion of enzyme identify will confirm the certainty of iTTP. On the other hand, enzyme deficiency in a patient lacking antibodies should trigger gene mutation studies to confirm cTTP. It is encouraging that the newest agent specific for TTP has been shown to be safe and effective in pediatric patients and can be used with confidence to help patients with iTTP. Research to better understand disease triggers, risk factors, and possibly susceptibility will aid in identifying those populations at greater risk while perhaps in the future lead to the design of interventions that prevent the condition from manifesting with its most dangerous complications.

## Figures and Tables

**Table 1:** Differential diagnosis of pediatric patients presenting with thrombocytopenia and signs of thrombotic macroangiopathic hemolytic anemia.

Diagnosis	cTTP	iTTP	ITP	HUS	DIC
Pathophysiology	Congenital deficiency of ADAMTS-13	Auto-antibodies against ADAMTS-13	Autoantibodies against platelet antigens	Shiga toxin-producing E.coli	Clotting factor consumption
ADAMTS-13 activity	Deficient/absent (<10%)	Deficient (<10%)	Normal	Minimally decreased	Decreased
Laboratory findings	↓ HGB, ↓ haptoglobin, ↑ LDH, ↑ indirect bilirubin, and severe thrombocytopenia (<20 × 109/L) with schistocytes Normal PT, PTT, and fibrinogen and a slight increase in D-dimer	↓ HGB, ↓ haptoglobin, ↑ LDH,↑ indirect bilirubin, and severe thrombocytopenia (<20 × 109/L) with schistocytes Normal PT, PTT, and fibrinogen and a slight increase in D-dimer	Isolated thrombocytopenia with normal HGB and WBC ↑LDH Normal PT, PTT, fibrinogen, and D-dimer	↓ HGB, ↓ haptoglobin, ↑ LDH,↑ indirect bilirubin, and thrombocytopenia with schistocytes Normal PT, PTT, and fibrinogen and a slight increase in D-dimer	↑ PT, ↑ PTT, ↑ D-dimer, ↓ fibrinogen, and thrombocytopenia with schistocytes
Treatment	Plasma infusion; recombinant human ADAMTS13; gene therapy	TPE (first line); immune suppression (steroids, rituximab); caplacizumab	Methylprednisolone; intravenous immunoglobin; thrombopoietin receptor agonists; rituximab; splenectomy	Supportive treatment; avoid antibiotics and antimotility agents; eculizumab	Treat the underlying cause; anticoagulation; cryoprecipitate; platelet transfusion; fibrinolytic therapy

Key to table abbreviations: cTTP: congenital thrombotic thrombocytopenic purpura; iTTP: immune-mediated thrombotic thrombocytopenia; ITP: immune thrombocytopenia; HUS: hemolytic uremic syndrome; DIC: disseminated intravascular coagulation, TPE: therapeutic plasma exchange.

## Data Availability

All relevant data are included in the manuscript.
